# A decade of “Chemical Frontiers Goa”

**DOI:** 10.1039/c8ra90068j

**Published:** 2018-08-14

**Authors:** Ramaswamy Murugavel, C. N. R. Rao

**Affiliations:** Department of Chemistry, Indian Institute of Technology Bombay Mumbai India-400076 rmv@chem.iitb.ac.in; New Chemistry Unit, Chemistry and Physics of Materials Unit, Sheikh Saqr Laboratory, International Centre for Materials Science, Jawaharlal Nehru Centre for Advanced Scientific Research (JNCASR) Jakkur P.O. Bangalore 560064 India cnrrao@jncasr.ac.in

## Abstract

We are delighted to present this collection of open access articles in RSC Advances to celebrate the Decennial Year of the “Chemical Frontiers Goa” meetings. 
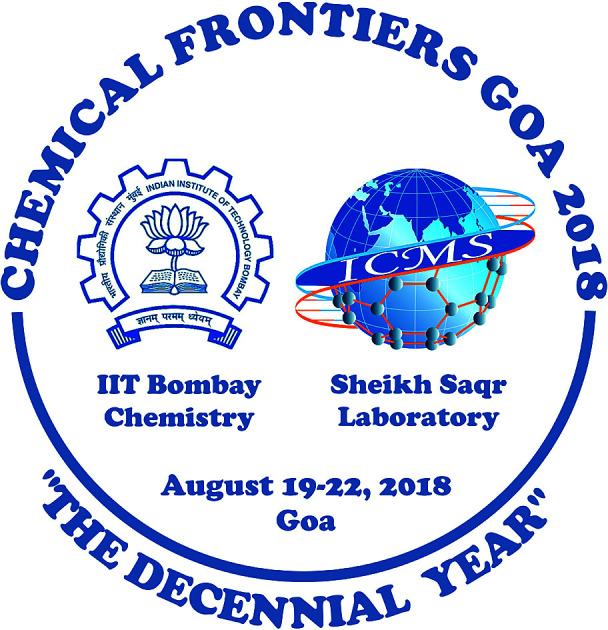

It is a matter of great pleasure for us to bring out this special issue of *RSC Advances* to celebrate the decennial year of the “Chemical Frontiers Goa” meeting in the chemical sciences.

This is an occasion to reminisce about the evolution of these meetings over the last ten years. The birth of this meeting took place when one of us (R. M) met the other (C. N. R. R) in the latter’s office in JNCASR Bangalore a little over 10 years ago, when C. N. R. R. emphasized the need to bring young chemistry researchers in India (along with some senior established chemists) together to a scenic location to discuss quality science. It was also suggested that in such meetings all the participants would stay in the same hotel for two to three days, eat meals together and more importantly attend lectures and plan new directions in chemistry. This idea took shape within a few months and we were indeed fortunate to see the first of the series of meetings happen during the monsoon of 2009 (August 14–16) in a beautiful location in South Goa (Holiday Inn Beach Resort). Due to the research interests of the two of us during that time, this meeting was aptly called “Chemistry of Functional Materials” (CFM), which continued with the same name for the first three editions. These initial meetings understandably included a good number of physicists, chemical engineers, material scientists, as well as chemists.

After the first three editions, the meeting was renamed as “Chemical Frontiers Goa” (CFG) so that the theme of the lectures delivered is more inclusive and includes organic chemists, biological chemists and spectroscopists, to name a few. This move rendered these meetings to become truly interdisciplinary, catalysing several new collaborative efforts between participants of different specializations. As time passed, the stature of CFG, as well as the interest among practicing chemists to attend this event, grew. We evolved a nomination system to select the speakers during recent years so that the early career researchers in many of the newer IITs and IISERs could showcase their important research. With ever-increasing interest in CFG, a cooling period generally applies to speakers who have already delivered a lecture, to get a chance to speak again.

In contrast to only a few student participants attending this meeting in the formative years, nowadays about 30–40% of participants are young Ph.D. students and post-doctoral fellows, who showcase their research findings in the form of either posters or short oral presentations. While in the earlier years, these students came mainly from the two organizing institutes (JNCASR and IIT Bombay), today they hail from several IITs, IISERs, national labs and universities. While a few aspects of CFG have changed over the years (*e.g.* the duration is now a day longer compared to the first five meetings), most other features remain the same. For example, CFG is yet to move out of South Goa, although it has moved around to different resorts in that region. The number of total participants of the meeting has been kept at around 100 all these years, so that the intensity of participation is not diluted.

We are happy that the high quality of the lectures has been maintained over the last decade. Significantly, several of the student participants of the initial meetings have now become faculty members and attend the meeting in their new role. It is our hope that CFG will be sustained in the coming decades with enhanced visibility.

To celebrate the decennial year, we thought that it would be appropriate to compile the highlights of Chemical Frontiers Goa as a collection of contributed peer-reviewed papers in a leading international journal. One of our partners over the years, Royal Society of Chemistry, readily agreed to publish such a special volume in *RSC Advances*. Equally enthusiastic were our past lecturers of CFG, who readily contributed articles covering important results of their recent research. This volume thus contains articles based on the lectures to be delivered by the invited speakers. It covers all areas of chemistry and showcases the variety of chemical research that is currently conducted in India.

We wish to thank Andrew Shore, Editor, *RSC Advances* and Rajesh Parishwad, RSC India, in collaborating with us in this effort and bringing out this collection in time for the tenth version of the “Chemical Frontiers Goa” meeting in August this year. We thank all the authors for accepting our request and contributing their articles on time to make this possible. We also place on record our sincere thanks to IIT Bombay and JNCASR Bangalore (particularly Sheikh Saqr Laboratory of ICMS) for their support of these meetings. Financial support received from the various sponsors over the years is also gratefully acknowledged. Several (co)conveners from IIT Bombay and JNCASR have helped organize this event over the last ten years and our special thanks are owed to them.

We sincerely hope that the readers of this special issue of *RSC Advances* will find the articles in this volume stimulating and useful for their research. We look forward to continued cooperation with all the partners of “Chemical Frontiers Goa” during the next decade and beyond.

## Supplementary Material

